# Opportunities for Guided Multichannel Non-invasive Transcranial Current Stimulation in Poststroke Rehabilitation

**DOI:** 10.3389/fneur.2016.00021

**Published:** 2016-02-24

**Authors:** Begonya Otal, Anirban Dutta, Águida Foerster, Oscar Ripolles, Amy Kuceyeski, Pedro C. Miranda, Dylan J. Edwards, Tihomir V. Ilić, Michael A. Nitsche, Giulio Ruffini

**Affiliations:** ^1^Neuroelectrics Barcelona, Barcelona, Spain; ^2^INRIA (Sophia Antipolis), Université Montpellier, Montpellier, France; ^3^University of Medicine Göttingen, Göttingen, Germany; ^4^Department of Radiology, Brain and Mind Research Institute, Weill Cornell Medical College, New York, NY, USA; ^5^Institute of Biophysics and Biomedical Engineering (IBEB), Faculdade de Ciências, Universidade de Lisboa, Lisboa, Portugal; ^6^Non-Invasive Brain Stimulation and Human Motor Control Laboratory, Burke-Cornell Medical Research Institute, White Plains, NY, USA; ^7^Department of Clinical Neurophysiology, Medical Faculty of Military Medical Academy, University of Defense, Belgrade, Serbia; ^8^Leibniz Research Centre for Working Environment and Human Factors, Technical University of Dortmund, Dortmund, Germany; ^9^Department of Neurology, University Medical Hospital Bergmannsheil, Bochum, Germany; ^10^Starlab Barcelona, Barcelona, Spain

**Keywords:** tDCS, non-invasive brain stimulation, transcranial current stimulation, multichannel stimulation, neuroimaging, near-infrared spectroscopy

## Abstract

Stroke is a leading cause of serious long-term disability worldwide. Functional outcome depends on stroke location, severity, and early intervention. Conventional rehabilitation strategies have limited effectiveness, and new treatments still fail to keep pace, in part due to a lack of understanding of the different stages in brain recovery and the vast heterogeneity in the poststroke population. Innovative methodologies for restorative neurorehabilitation are required to reduce long-term disability and socioeconomic burden. Neuroplasticity is involved in poststroke functional disturbances and also during rehabilitation. Tackling poststroke neuroplasticity by non-invasive brain stimulation is regarded as promising, but efficacy might be limited because of rather uniform application across patients despite individual heterogeneity of lesions, symptoms, and other factors. Transcranial direct current stimulation (tDCS) induces and modulates neuroplasticity, and has been shown to be able to improve motor and cognitive functions. tDCS is suited to improve poststroke rehabilitation outcomes, but effect sizes are often moderate and suffer from variability. Indeed, the location, extent, and pattern of functional network connectivity disruption should be considered when determining the optimal location sites for tDCS therapies. Here, we present potential opportunities for neuroimaging-guided tDCS-based rehabilitation strategies after stroke that could be personalized. We introduce innovative multimodal intervention protocols based on multichannel tDCS montages, neuroimaging methods, and real-time closed-loop systems to guide therapy. This might help to overcome current treatment limitations in poststroke rehabilitation and increase our general understanding of adaptive neuroplasticity leading to neural reorganization after stroke.

## Introduction

Although spontaneous poststroke recovery occurs, between 15% and 30% of stroke survivors are left permanently disabled (1). Poststroke rehabilitation helps relearn skills that are lost when part of the brain is damaged. As an adjunct therapy, non-invasive brain stimulation (NIBS) techniques, including repetitive transcranial magnetic stimulation (rTMS) and transcranial current stimulation (tCS) – particularly direct current stimulation (tDCS) – are promising approaches to enhance the effects of standardized rehabilitation treatments in selected poststroke patients. Like rTMS, tDCS can alter cortical excitability in predictable ways. tDCS is characterized as neuromodulatory rather than neurostimulatory, since the currents delivered during tDCS are not sufficient to directly generate action potentials. tDCS-induced excitability alterations depend on the duration, current density, and direction of the current flow. Generally, anodal tDCS (a-tDCS) enhances excitability, while cathodal tDCS (c-tDCS) reduces it (2–4). Whereas after-effects of single stimulation sessions are in the time range of early-phase long-term potentiation and long-term depression (~1 h), repetitive stimulations with certain intervals can induce late-phase effects lasting longer than 24 h after intervention (3–6). tDCS is a well-tolerated technique, easily applied over cortical targets leading to adaptive neural reorganization and the reduction in maladaptive plasticity during behavioral treatment. Further, tDCS is less expensive and likely to be better accepted by patients than rTMS (7, 8), making it potentially well poised for home therapy.

Currently, the need to target not an isolated cortical region, but several functionally correlated cortical hubs involved in larger scale intrinsic brain networks is becoming increasingly recognized (9, 10). Advances in neuroimaging technology, such as functional magnetic resonance imaging (fMRI), diffusion tensor imaging, electroencephalography (EEG), and functional near-infrared spectroscopy (fNIRS), are allowing us to non-invasively visualize and quantify brain network connectivity in humans with increasing accuracy. Recently, we showed how the optimal electrode configuration of a multichannel tDCS system can be determined by using neuroimaging data to specify a target map on the cortical surface for excitatory or inhibitory stimulation (11). Multichannel tDCS is a new approach highly capable of efficiently targeting distributed brain networks to facilitate beneficial neuroplasticity and functional connectivity leading to poststroke recovery.

Portable neuroimaging solutions, such as EEG and fNIRS, can objectively capture individual brain states poststroke, which can be used to customize and adapt NIBS in real time to facilitate training (12, 13). An EEG–fNIRS-based method (14) was recently proposed for screening and monitoring of neurovascular coupling functionality in combination with tDCS. In this system, neuronal and hemodynamic responses were abstractly represented as feedback for tDCS effects. Such innovative portable EEG–fNIRS neuroimaging systems could be used to objectively guide and quantify the progress of a tDCS treatment regime in conjunction with neurorehabilitation. Moreover, system identification and parameter estimation techniques using neuronal and hemodynamic responses to tDCS can be used to track the effects, e.g., on corticospinal excitability, for closed-loop control of tDCS. Poststroke integrity of task-specific ipsilesional and/or contralesional neural pathways can be determined with EEG–fNIRS neuroimaging during task performance, which can be leveraged toward the optimization of subject-specific tDCS. The goal may be to correlate functional outcome with regard to EEG–fNIRS brain activation patterns as a marker of the underlying task-specific residual activation such that those residual brain activation patterns are facilitated with individualized brain state-dependent multichannel tDCS as an adjunct treatment during stroke rehabilitation.

Here, we introduce the potential of these two methods (11, 14) to optimize future multichannel tDCS systems for modulation of excitability of brain networks, represented by spatially extended cortical targets. Combining both models closely addresses the individual determinants of patterns of neuroplastic changes both to guide tDCS treatment and to assess functional recovery. We present potential novel application opportunities based on guided multichannel tDCS in poststroke rehabilitation. Likewise, we show how multimodal approaches pairing neuroimaging and electrophysiological measures with therapeutic tDCS can extend its potential in aiding customized and personalized long-term rehabilitation strategies, including post-acute rehabilitation after stroke.

## tDCS-Based Poststroke Neurorehabilitation

Poststroke functional recovery depends on the degree of adaptive neuroplasticity in central nervous system reorganization. Adaptive neuroplasticity includes changes in synaptic connectivity and excitability in surviving neural cell population in the perilesional zone, in remote structures, and in the contralateral unaffected hemisphere in case of a mono-hemispheric lesion (15, 16). In particular, mono-hemispheric stroke is thought to result in disinhibition of the contralesional unaffected hemisphere due to release from transcallosal inhibition. This may exert an inhibitory influence on perilesional areas, negatively affecting spontaneous neuroplasticity and interfering with the ability of perilesional areas to contribute to functional recovery – with the exception of particular cases with extensive stroke lesions. This interhemispheric inhibition model provides the rationale for facilitatory stimulation of the peri-stroke areas (hypoactive cortical regions), and suppression of the contralesional hemispheric hyperactivity with NIBS in order to enhance functional performance in poststroke patients (17). Further, a recent bimodal balance-recovery model links interhemispheric balancing and functional recovery to the structural reserve spared by the stroke lesion (18). This new concept raises the question about optimal localizations and number of positions to be stimulated with tDCS.

Some meta-analyses evaluated the efficacy of tDCS on poststroke rehabilitation for limb motor impairments, impaired balance, hemineglect, aphasia, and dysphagia. In Table 1, we summarize studies extracted from recent meta-analyses and systematic reviews that satisfy the following four criteria. They were randomized controlled trials (RCTs) or randomized cross-over trials (with sham controls), with mono-hemispheric poststroke adult patients (no chronicity limits), that received tDCS combined with standardized therapy, and reported outcome measures.

**Table 1:** **Summary of tDCS-based poststroke neurorehabilitation studies**.

Study	Study design		Parameters of stimulation				Location		Combined therapy		Effect (+/Nd)
Reference	Subjects	Stroke phase	Stimulation	Current intensity, current density	Duration day (min)	Num. sessions	Target electrode region	Reference region	Therapy type	Online/offline	A+/B+/C+/S+
**Upper limb motor impairment**											
Bolognini et al. (19)	14	Chr.	B/S	2 mA, 0.057 mA/cm^2^	40	10	A: affected M1, C: unaffected M1	Unaffected M1	Constraint-induced movement therapy	Online	B+ motor performance
Celnik et al. (20)	9	Chr.	A/S	1 mA, 0.11 mA/cm^2^	20	4	Affected M1 (abductor pollicis brevis muscle hot-spot)	Contralateral supraorbital area	Peripheral nerve stimulation	Offline	A+
Di Lazzaro et al. (21)	20	Acu.	B/S	2 mA, 0.057 mA/cm^2^	40	5	A: affected M1, C: unaffected M1 (abductor pollicis brevis muscle hot-spot)	Unaffected M1	Constraint-induced movement therapy	Online	Nd – but B reduces II
Fregni et al. (22)	6	Chr.	A/C/S	1 mA, 0.028 mA/cm^2^	20	3	A: affected M1, C: unaffected M1 (first dorsal interosseous muscle hot-spot)	Contralateral supraorbital area	JTT	Online	A+; C+
Fusco et al. (23)	11	Acu.	C/S	1.5 mA, 0.043 mA/cm^2^	10	10	Unaffected M1	Right shoulder	Traditional motor rehabilitation	Offline	Nd
Hesse et al. (24)	96	Acu. Sub.	A/C/S	2 mA, 0.057 mA/cm^2^	20	30	A: affected M1, C: unaffected M1	Contralateral supraorbital area	Robotic arm training	Online	A+; C+
Khedr et al. (25)	40	Sub.	A/C	2 mA, 0.057 mA/cm^2^	25	6	A: affected M1, C: unaffected M1	Contralateral supraorbital area	Inpatient daily rehabilitation	Offline	A+; C+
Kim et al., 2009 (26)	10	Sub.	A/S	1 mA, 0.04 mA/cm^2^	20	2	Affected M1 (first dorsal interosseous muscle hot-spot)	Contralateral supraorbital area	Box and block test; Finger acceleration	Online	A+
Kim et al. (27)	18	Sub.	A/C/S	2 mA, 0.08 mA/cm^2^	20	10	A: affected M1, C: unaffected M1 (first dorsal interosseous muscle hot-spot)	Contralateral supraorbital area	Conventional occupational therapy	Online	C+
Lefebvre et al. (28)	18	Chr.	B/S	1 mA, 0.028 mA/cm^2^	30	2	A: affected M1 (hand muscle hot-spot)	C: unaffected M1 (hand muscle hot-spot)	Motor skill learning task	Online	B+
Lefebvre et al. (29)	19	Chr.	B/S	1 mA, 0.028 mA/cm^2^	30	2	A: affected M1 (hand muscle hot-spot)	C: unaffected M1 (hand muscle hot-spot)	Motor skill learning task	Online	B+
Lindenberg et al. (30)	20	Chr.	B/S	1.5 mA, 0.092 mA/cm^2^	30	5	A: affected M1	C: unaffected M1	Conventional physical and occupational therapy	Online	B+
Mortensen et al. (31)	15	Chr.	A/S	1.5 mA, 0.04 mA/cm^2^	20	5	Affected M1	Contralateral supraorbital area	Conventional occupational therapy	Online	A+
Nair et al. (32)	14	Chr.	C/S	1 mA/?	30	5	Unaffected M1	Contralateral supraorbital area	Conventional occupational therapy	Online	C+
Rocha et al. (33)	21	Chr.	A/C/S	2 mA, 0.057 mA/cm^2^	A: 13 C: 9	12	A: affected M1, C: unaffected M1	Contralateral supraorbital area	Constraint-induced movement therapy (modified)	Offline	A+; C+
Triccas et al. (34)	23	Sub. Chr.	A/S	1 mA, 0.028 mA/cm^2^	20	18	Affected M1	Contralateral supraorbital area	Robotic therapy	Online	Nd
Viana et al. (35)	20	Chr.	A/S	2 mA, 0.057 mA/cm^2^	13	15	Affected M1	Contralateral supraorbital area	Virtual reality therapy	Offline	Nd (A+; S+)
Wu et al. (36)	90	Sub. Chr.	C/S	1.2 mA, 0.26 mA/cm^2^	20	20	Affected primary sensorimotor cortex	Unaffected shoulder	Conventional physical therapy	Offline	C+
**Lower limb motor impairment and poor balance**											
Chang et al. (37)	24 (12/12)	Acu.	A/S	2 mA, 0.28 mA/cm^2^	10	10	Affected tibialis anterior muscle hot-spot	Contralateral supraorbital area	Conventional physical therapy	Online	A+
Madhavan et al. (38)	9	Chr.	A/S	0.5 mA, 0.06 mA/cm^2^	15	3	Unaffected and affected lower limb primary motor cortex	Contralateral supraorbital area	Tracking task sinusoidal waveform	Online	A+
Sohn et al. (39)	11	Sub.	A/S	2 mA, 0.08 mA/cm^2^	10	2	Affected quadriceps femoris muscle hot-spot	Contralateral supraorbital area	Standard rehabilitation	Offline	A+
Tanaka et al. (40)	8	Chr.	A/S	2 mA, 0.057 mA/cm^2^	10	2	Affected tibialis anterior muscle hot-spot	Contralateral supraorbital area	Knee extension task	Online	A+
**Hemineglect**											
Ko et al. (41)	15	Sub.	A/S	2 mA, 0.08 mA/cm^2^	20	2	Right PPC	Contralateral supraorbital area	No	Offline	A+
Làdavas et al. (42)	30	Sub. Chr.	A/C/S	2 mA, 0.057 mA/cm^2^	20	10	A: right PPC C: left PPC	Contralateral supraorbital area	Prism adaptation treatment	Online	A+ > C+
Sparing et al. (43)	10	Sub. Chr.	A/C/S	1 mA, 0.04 mA/cm^2^	10	3	A: right PPC and left PPC, C: left PPC	Cz	No	Offline	A+ over r-PPC; C+
Sunwoo et al. (44)	10	Sub. Chr.	A/B/S	1 mA, 0.04 mA/cm^2^	20	3	A: right PPC, C: left PPC	Contralateral supraorbital area	No	Offline	B+ > A+
**Aphasia**											
Fiori et al. (45)	7	Chr.	A/S	1 mA, 0.028 mA/cm^2^	20	10	Broca’s and Wernicke’s area	Contralateral frontopolar cortex	Video naming	Online	A+
Flöel et al. (46)	12	Chr.	A/C/S	1 mA, 0.028 mA/cm^2^	40	3	Right temporo-parietal cortex	Contralateral supraorbital area	Picture naming	Online	A+ > C+
Kang et al. (47)	10	Chr.	C/S	2 mA, 0.08 mA/cm^2^	20	5	Right Broca’s homolog	Contralateral supraorbital area	Picture naming	Online	C+
Marangolo et al. (48)	8	Chr.	B/S	2 mA, 0.057 mA/cm^2^	20	10	A: Broca’s area	C: right Broca’s homolog	Word repetition training	Online	B+
Monti et al. (49)	9	Chr.	A/C/S	2 mA, 0.057 mA/cm^2^	10	10	Broca’s area	Right shoulder	Picture naming	Online	C+
You et al. (50)	21	Sub.	A/C/S	2 mA, 0.057 mA/cm^2^	30	10	A: Wernicke’s area, C: right Wernicke’s homolog	Contralateral supraorbital area	Auditory verbal comprehension	Offline	C+ > A+
**Dysphagia**											
Kumar et al. (51)	14	Sub.	A/S	2 mA, 0.13 mA/cm^2^	30	5	Unaffected swallowing motor cortex	Contralateral supraorbital area	SSM	Online	A+
Shigematsu et al. (52)	20	Chr.	A/S	1 mA, 0.028 mA/cm^2^	20	10	Affected pharyngeal motor cortex	Contralateral supraorbital area	SSM	Online	A+
Yang et al. (53)	14	Sub.	A/S	1 mA, 0.04 mA/cm^2^	20	10	Affected pharyngeal motor cortex	Contralateral supraorbital area	SSM	Online	A+ only after follow-up

*Studies have been extracted from recent meta-analyses following aforementioned criteria*.

*+, improves the effect on task performance after tDCS intervention; ?, not mentioned; A, a-tDCS; Acu., acute; B, bilateral a- and c-tDCS; C, c-tDCS; Chr., chronic; II, interhemispheric imbalance; JTT, Jebsen–Taylor Hand Function Test; M1, primary motor cortex; Nd, no differences; PPC, posterior parietal cortex; S, sham-tDCS; SSM, standardized swallowing maneuvers; Sub., subacute*.

### Upper Limb Motor Impairment

More than 50% of stroke survivors exhibit some degree of motor impairment and require partial assistance in activities of daily living (ADL). Restriction of the upper limb motor function can limit ADL performance, directly influencing quality-of-life. Most tDCS interventional trials with poststroke patients with arm and hand impairments showed favorable results on upper limb motor function (Table 1), influencing muscle strength (31) and muscle tone (36), too. Yet, combination of tDCS with robotic (34) or virtual reality therapy (35) was not found to improve the effect of recovery outcome. According to some systematic reviews (54–56), the effectiveness of tDCS on upper limb motor function varies, showing only small to moderate effects. A dose–response relationship was observed (57) between upper extremity motor recovery and application of tDCS dependent on electrode size, charge density, and current density.

### Lower Limb Motor Impairment and Poor Balance

Gait impairment includes speed, endurance, and stability. About 57–63% of stroke survivors cannot walk independently at symptom onset and 22–50% after gait rehabilitation (58). Most studies using NIBS focused on the recovery of the paretic upper extremity rather than on the recovery of lower limb function and balance. This might be due to functional and anatomical limitations, since the lower limb motor cortex and the cerebellum may not be easily reached by NIBS techniques. Nevertheless, some studies demonstrated that tDCS protocols are able to enhance lower limb motor function (37, 38), motor cortex excitability (37), and muscle strength (39, 40) (Table 1). Considering balance impairment, static postural stability was observed to significantly improve after tDCS (39, 59). Some studies have investigated the effect of cerebellar tDCS on balance in healthy subjects and neurological patients (e.g., Parkinson), but no study so far evaluated the effects of cerebellar tDCS on poor balance in poststroke patients.

### Hemineglect

Patients suffering from hemineglect do not attend or respond to information on the contralesional side of space (60). Several studies indicate that poststroke hemineglect constitutes a predictive factor of poor functional prognosis (61–63). In recent studies of hemineglect rehabilitation (16), tDCS was applied to either facilitate the right (affected) or suppress the left (unaffected) posterior parietal cortex (PPC) activation, based on the aforementioned interhemispheric inhibition model. Accordingly, a-tDCS over the right PPC was shown to improve hemineglect rehabilitation (41–44) and c-tDCS over the left PPC to reduce hemineglect symptoms (43). Further, bilateral tDCS is considered to induce stronger effects (44) (Table 1).

### Aphasia

Even after speech and language therapy, 12% of poststroke survivors are left with some degree of chronic communication deficit (64–66). Following the model of interhemispheric inhibition, c-tDCS over right-hemispheric Broca’s homolog was demonstrated to significantly improve naming accuracy (47). Bihemispheric a-tDCS over left Broca’s area and c-tDCS over right Broca’s homolog was suggested to improve recovery in different language tasks (48). In contrast, a-tDCS over the right temporoparietal cortex was shown to stronger enhance the overall training effect in naming ability in comparison to c-tDCS (46). These and other tDCS trials [(45–50), Table 1] were evaluated in a meta-analysis (67) showing a moderate, but non-significant, pooled size effect favoring tDCS. This might be due to heterogeneity among study protocols and insufficient numbers of study subjects, since all trials favored tDCS. When pooling rTMS and tDCS studies that suppress activation of right-hemispheric homologous language regions, a significant effect of the intervention was identified (68).

### Dysphagia

At least one out of two stroke patients experiences swallowing problems (69, 70) or dysphagia, which is potentially fatal. Recently, two meta-analyses (71, 72) have independently evaluated the effects of NIBS (rTMS and tDCS) on dysphagia with the aim to encourage more efficient rehabilitation techniques. Both meta-analyses included the same three tDCS studies (51–53). All three studies used a-tDCS, but two studies stimulated the affected hemisphere (52, 53), whereas one study stimulated the unaffected hemisphere (51) (Table 1). There was a moderate, but non-significant, pooled size effect favoring tDCS intervention vs. sham-tDCS. When pooling rTMS and tDCS studies together, a meta-analysis (71) found that stimulating the unaffected hemisphere improved swallowing significantly. Bilateral tDCS – though not explored so far – might be especially attractive for dysphagia therapy because swallowing is bilaterally innervated.

## Multichannel tDCS for Distributed Cortical Targets

Heterogeneous statements about efficacy of tDCS are highly likely to be due to the variability of study protocols and the limited number of participants. Stroke is a heterogeneous disease with regard to lesion size and location requiring customized rehabilitation strategies to result in optimal effects. Here, we present methods to tackle this issue, such as a methodological tool for optimizing multichannel tDCS montages and efficiently targeting complex, distributed cortical areas (11). With a constraint on the maximal number of electrodes and currents, an optimal multichannel tDCS montage solution (electrode currents and locations) can be obtained by using neuroimaging data. The present implementation of this method (*Stimweaver*) relies on the fast calculation of multichannel tDCS electric fields (including components normal and tangential to the cortical boundaries) using a five-layer finite element model of a realistic head (73). Solutions are found using constrained least squares to optimize current intensities, with electrode number and location selected using a genetic algorithm.

A key aspect is the definition of the problem to be optimized. This is done by specifying two cortical surface maps. The first map provides a target electric field – in the form of the electric field component normal to the cortical surface – at each location (e.g., *E*_n_ = −0.25 V/m in region 1 and *E*_n_ = +0.25 V/m in region 2). Our current understanding of the effects of tDCS focuses on the orientation of the electric field in relation to the orientation of neurons, and in particular, pyramidal neurons in the cortex (11, 74). Based on the approximation that the effects of current stimulation are due to the linear interaction of electric fields with populations of elongated cortical neurons, we argue that the optimization problem for tDCS can be defined in terms of the component of the electric field orthogonal to the cortical surface (generally, the same methodology and logic applicable to TMS). According to this model, “inward” directed fields are excitatory and “outward” directed fields inhibitory. This (signed) *target map* may specify several discrete areas or just a continuous function. The second map is a cortical *weight map* taking positive values, specifying the importance of each target area with a weight. This methodology is particularly appropriate for the neuromodulation of cortical (distributed) networks. In the following sections, we provide two examples of the application of this methodology to the case for poststroke network modulation.

### Multichannel tDCS for Poststroke Lower Limb Motor Rehabilitation

**Figure 1:**
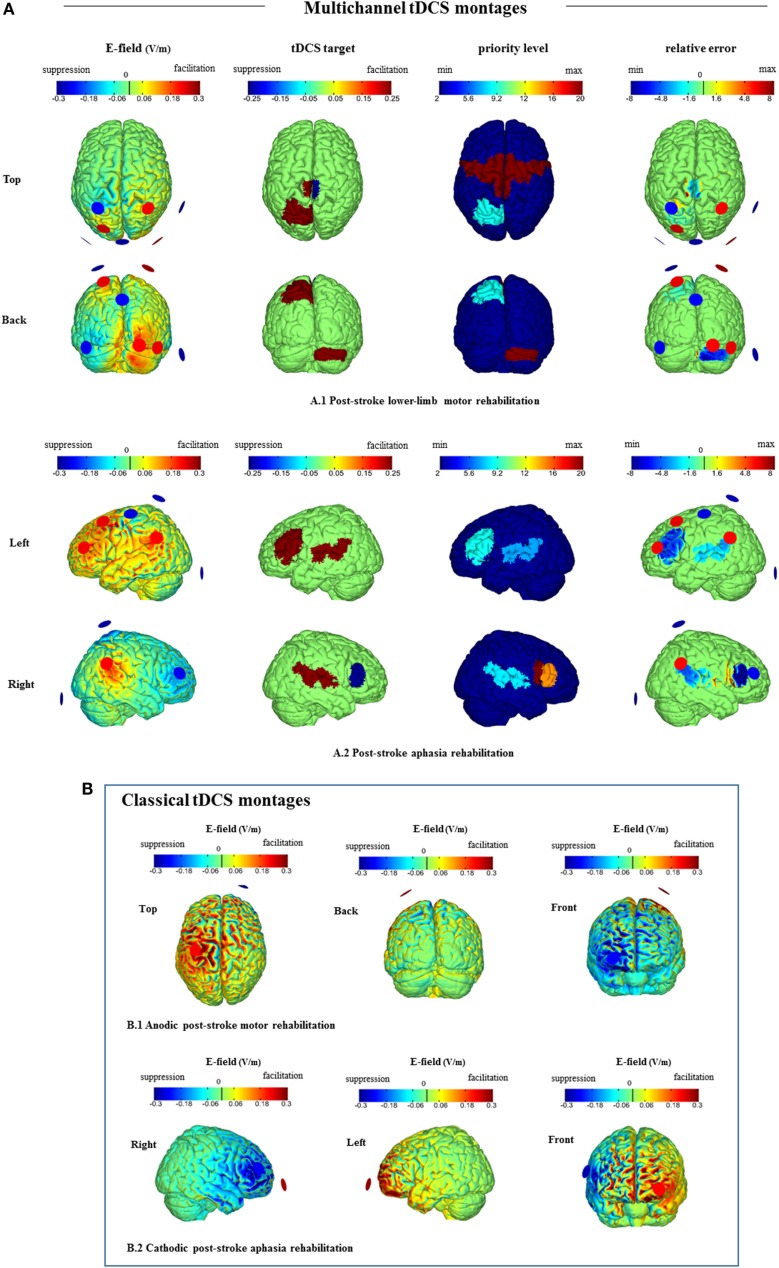
***Stimweaver* simulations for (A) guided multichannel tDCS montages vs. (B) classical tDCS montages**. **(A)** Multichannel tDCS representations for distributed cortical targets for **(A.1)** poststroke lower limb motor rehabilitation (top and back views, see Multichannel tDCS for Poststroke Lower Limb Motor Rehabilitation) and **(A.2)** poststroke aphasia rehabilitation (left and right views, see Multichannel tDCS for Poststroke Aphasia Rehabilitation). Optimal solution using eight Neuroelectrics Pistim circular electrodes (1 cm radius and Ag/Cl). Total injected current 4 mA. Plots of the normal component of the *E-field* (V/m) (left), *tDCS target* region (center left), *priority level* (center right), and *relative error* (right) shown on the gray matter. In the left column, positive (red) colors reflect ingoing, excitatory normal electric fields (blue the opposite). In the second column, red areas denote targets to facilitate activation and blue to suppress activation. The third column colors reflect the importance (*weight*) of each area taking positive values up to 20. A dark blue cortical area reflects minimum/default priority and a red area maximum priority. In-between colors denote the corresponding intermediate priority. The last column provides a visual display of the match of electric fields solution to target [the *relative error* (10)]. Note that this model may not fit each poststroke patient with lower limb **(A.1)** or language **(A.2)** impairment because areas important for restitution are likely to be different according to lesion size and location (see Multichannel tDCS for Poststroke Lower Limb Motor Rehabilitation and Multichannel tDCS for Poststroke Aphasia Rehabilitation for details). **(B)** Plots of the normal component of the E-field (volts per meter) of classical tDCS montages for **(B.1)** anodic poststroke motor rehabilitation (top, back, and frontal views) and **(B.2)** cathodic poststroke aphasia rehabilitation (left, right, and frontal views). Solutions using two Neuroelectrics Pistim circular electrodes. Total injected current 2 mA. **(B.1)** Anodic stimulation over the M1 affected area: “active” electrode on C1 and cathode (return electrode) over the contralateral supraorbital area (38). **(B.2)** Cathodic stimulation over the right homolog of Broca’s area: “active” electrode on F6 and anode (return electrode) over the contralateral supraorbital area (47).

For development of multichannel tDCS on a rational basis, identification of areas relevant for rehabilitation of motor function after stroke is crucial. Following recent longitudinal studies after stroke, Figure 1A.1 targets at tDCS-induced facilitatory activity on the ipsilesional primary sensorimotor cortex (75, 76) and on contralesional cerebellum (77), which are areas associated with functional improvement. Based on a recent review (8), we also aim to upregulate excitability of ipsilesional M1 and to downregulate excitability of contralesional M1 (Figure 1A.1). For the premotor cortex (PMC), some functional brain-imaging studies have demonstrated increased activation of the ipsilesional area during movement of the affected limb after stroke (78). This activity improvement might however be dysfunctional, since the inhibitory function of the PMC was found disturbed in stroke patients with poor motor function (79). Takeuchi et al. (80) hypothesized that disinhibition of the ipsilesional PMC causes a dysbalanced activity distribution of motor cortex proximal limb representations, which results in a proximal-dominant competitive interaction between ipsilesional M1 and PMC. This widespread maladaptive activity will result in poor control of the paretic distal parts of the limb in stroke patients. To avoid such widespread disinhibition of motor-related areas that may lead to maladaptive plasticity, we attributed a high priority to “no stimulation” of the PMC in the first row of Figure 1.

### Multichannel tDCS for Poststroke Aphasia Rehabilitation

Recent neuroimaging studies on poststroke aphasia reveal neuroplastic cortical changes in both hemispheres, yet how the areas of the structural and functional language neural network contribute to language relearning success is still controversial (81). An optimized multichannel tDCS montage may allow us to facilitate and suppress activation of specifically selected language-relevant cortical areas (82, 83) aiming at individualizing optimal parameters for each poststroke aphasic patient in the near future. Figure 1A.2 presents the results of a generalized montage targeting those areas of the language network, in which tDCS had a beneficial effect on language functions in previous studies (Table 1). To improve naming ability, we target to suppress right pars triangularis activation over Broca’s homolog (47, 84), but try to minimize stimulation over the right pars opercularis (84). Further, we induce facilitatory tDCS over left Broca’s and Wernicke’s areas (45), and also over the right temporoparietal cortex (46).

## Real-Time fNIRS–EEG Feedback for Multichannel tDCS

During neural activation, the electric currents from excitable membranes of brain tissue superimpose in the extracellular medium and generate a potential on the scalp (i.e., EEG). Neural activity has been shown to be closely related, spatially and temporally, to cerebral blood flow (CBF) that supplies glucose *via* neurovascular coupling. The hemodynamic response can be captured by fNIRS, which enables continuous monitoring of cerebral oxygenation and blood volume. This neurovascular coupling phenomenon led to the concept of the neurovascular unit (NVU), which consists of the endothelium, glia, neurons, pericytes, and the basal lamina. Recent work suggests NVU as an integrated system working in concert using feedback mechanisms to enable proper brain homeostasis and function. Capturing these mostly non-linear spatiotemporal interactions within NVU remains a challenge (85). The dynamic nature of functional connectivity may be in part due to spatiotemporal interactions between neuro- and hemodynamics. We postulate that fusing of EEG and fNIRS data provides a more robust real-time tracking of the dynamic functional connectivity during task performance. Further, multivariate machine learning methods (86) can be leveraged for fusing multimodal functional neuroimaging data.

In EEG and fNIRS, the sensors measuring brain activity are located outside of the head, thus a source space representation of the sensor readings (inverse problem) has to be inferred from a physical model that maps cortical (source) activity to the sensors (forward problem). MRI-based fast individualized quasistatic bioelectromagnetic forward simulations can be performed using open source software developed for EEG analysis [e.g., OpenMEEG (87)]. In the case of fNIRS, the MRI-based individualized physical model involves optical properties, such as absorption and scattering coefficients of different tissue types, and describes the photon transport through the tissue (88). Diffuse optical tomography (89) extends fNIRS by applying overlapping “high density” measurements, thus providing three-dimensional imaging with improved spatial resolution. In the source space, the multimodal (EEG–fNIRS) functional connectivity needs to be analyzed in real time for closed-loop control of multichannel tDCS.

Computational methods to investigate the dynamic functional connectivity in the source space can be developed based on prior works in fMRI–EEG (90, 91). Moreover, for motor tasks, sparse linear regression analysis has been shown to be well suited to reconstruct the electromyogram from human cortical activations using fMRI (92) and EEG (93). Thus, multichannel tDCS systems (11) can be optimized to target relevant cortical activations (source space) – related to functional connectivity hubs – found from EEG–fNIRS joint neuroimaging during task performance to facilitate poststroke rehabilitation (94). The real added value to the neuroimaging-guided multichannel tDCS paradigm is that EEG–fNIRS data can be used to individualize NIBS protocols not only based statically on the “hot spots” of beneficial neuroplasticity in a given patient but also to adjust stimulation protocols dynamically based on physiological feedback to approach optimal activation of the target regions in the time domain.

## Conclusion

Transcranial direct current stimulation is a promising evolving adjunctive therapy in stroke rehabilitation based on enhancement of beneficial and reduction of maladaptive plasticity. However, the current state of the art suffers from relevant limitations, which hinder the full integration of tDCS methodology in clinical practice. First, small sample sizes, heterogeneity of samples (e.g., lesion type, poststroke severity, and time after stroke), and differences between stimulation protocols might be the main reasons for so far limited magnitude of tDCS effects and heterogeneity of results. Second, efficacy of stimulation might be relevantly limited by current restriction to stimulation of one or two target areas in most studies, not taking into account the complex networks involved in respective functions. Third, in a disease characterized by heterogeneous lesions, largely ignoring individual differences of lesion size, location, and baseline activation, as well as anatomical factors relevant for the efficacy of intervention, e.g., head size and brain anatomy, might furthermore limit the efficacy of current stimulation approaches. Larger controlled studies are necessary to determine the best parameters of stimulation (including the optimal cortical target locations) according to each subtype of stroke, the time course of stroke recovery, and individual factors. With the introduction of neuroimaging-guided multichannel tDCS protocols, most of these problems may be solved, and these approaches might relevantly help to determine the real potential of tDCS to improve clinical symptoms after stroke.

## Author Contributions

BO, AD, AF, PM, MN, and GR have substantially contributed to the conception, design, and interpretation of data for this work. OR has substantially contributed to the analysis and simulations of this work. DE, AK, and TI have substantially contributed to the interpretation of data. All authors have also drafted the work and revised it critically with contribution related to author order. All authors have approved the final version prior to submission and are in agreement.

## Disclaimer

The content is solely the responsibility of the authors and does not necessarily represent the official views of the National Center for Advancing Translational Science or the National Institutes of Health.

## Conflict of Interest Statement

GR is co-owner of Neuroelectrics and Starlab and holds patents on multisite tCS. PM and MN are members of Neuroelectrics’ advisory board.
